# Risky sexual behaviours and HIV testing among young people in Ghana: evidence from the 2017/2018 Multiple Indicator Cluster Survey

**DOI:** 10.1186/s12978-022-01439-1

**Published:** 2022-05-28

**Authors:** Adom Manu, Deda Ogum-Alangea, Joshua Cobby Azilaku, Emmanuel Anongeba Anaba, Kwasi Torpey

**Affiliations:** 1 Department of Population, Family and Reproductive Health, School of Public Health, College of Health Sciences, University of Ghana, Accra, Ghana; 2 College of Health and Wellbeing, Kintampo, Ghana

**Keywords:** Risky sexual behaviour, Young people, HIV testing, Ghana, Condom use

## Abstract

**Background:**

Young people have a higher chance of experimenting with sex before marriage, thus they engage in risky sexual behaviours that predispose them to HIV infections. The objective of this study was to assess the relationship between engaging in risky sexual behaviours and the uptake of HIV testing services among young people in Ghana.

**Methods:**

We analysed secondary data from the 2017/2018 Ghana Multiple Indicator Cluster Survey, which collected data on population and health indicators across the previous ten regions of Ghana, using a Computer Personal Assisted Interviewing application. Data were analysed using descriptive statistics, Chi-square test and Binomial Logistic regression.

**Results:**

Seventy-nine per cent (79%) of young women and 68% of young men did not use a condom during last sexual intercourse. In addition, 68% of young women and 87% of young men had not tested for HIV. Young women (AOR = 2.19; 95% CI 1.56–3.07) and young men (AOR = 3.38; 95% CI 1.18–9.64) aged 20–24 years had a higher likelihood of being tested for HIV compared to those aged 15–19 years. Young women with junior high school education (AOR = 2.03; 95% CI 1.08–3.81) were more likely to test for HIV compared with those who had pre-primary/no formal education. In addition, young women who were never married or in a union (AOR = 0.39; 95% CI 0.27–0.56) had 61% of reduced odds of being tested for HIV compared with those who were currently married or in a union. There was no significant association between risky sexual behaviours and HIV testing (p > 0.05).

**Conclusion:**

This study demonstrated that condom use among sexually active young people was low. The uptake of HIV testing services was below expectation. Age, educational status, marital status and exposure to the mass media were the salient factors influencing the uptake of HIV testing among young people. Stakeholders should implement interventions to help increase the uptake of HIV testing and condom use among young people in Ghana.

## Plain Language summary

Sub-Sahara Africa is the only region in the world where the number of young people continues to increase greatly. Many of the new HIV infections among young people across the world happen in sub-Saharan Africa. Young people engage in unsafe sexual behaviours, such as having sex without a condom, beginning sexual intercourse at an early age and having sex with many partners, which increases their chance of getting infected with HIV. Testing for HIV helps in the early discovery of the virus which is crucial for effective HIV management and prevention. Increasing HIV testing is one of the health issues cherished by global health leaders. However, little is known about whether young people who engage in unsafe sexual behaviours utilize HIV testing services. The analysed data were collected as part of the 2017/2018 Multiple Indicator Cluster Survey across all the regions of Ghana. Our findings showed that many of the sexually active young people who participated in the survey had not tested for HIV, hence did not know their HIV status. Making matters worse, many of them engaged in unsafe sexual behaviours, such as unprotected sexual intercourse. Young people aged 20–24 years, those with higher education and married young women were more likely to utilize HIV testing services. To help end HIV as a public health threat by 2030, it is very necessary for stakeholders to educate young people about the dangers associated with unsafe sexual behaviours as well as encourage them to know their HIV status.

## Background

Young people are persons between the ages of 10 and 24 years [1]. Globally, sub-Sahara Africa (SSA) is the only region where the population of young people continues to grow considerably [2]. Young people have a higher likelihood of experimenting with sex before marriage, hence they involve in sexual risk behaviours, such as multiple sexual partners, unprotected sexual intercourse, early sexual initiation and casual sex [3, 4]. In SSA, the median age of sexual debut ranges between 16–18 years, yet it is one of the regions with low contraceptive uptake among young people. In Ghana, risky sexual behaviours among young people are common. In 2020 alone, 110,000 teenage pregnancies occurred in the country, equivalent to 301 pregnancies every day and 31 pregnancies every hour [5]. This is evident that young people in Ghana are sexually active and engage in unprotected sexual intercourse [6]. Risky sexual behaviours are associated with being male, being single, being employed, having lower educational status, using substances and alcohol and watching pornographic videos [4, 7].

Young people who engage in risky sexual behaviours are predisposed to several sexual and reproductive health problems, including sexually transmitted infections such as HIV [3, 8]. Although the prevalence of HIV/AIDS has reduced worldwide, approximately 50% of all HIV incidences occur among young people. A substantial proportion (more than 80%) of the new HIV cases occur among young people in SSA [8]. As of 2014, the national prevalence of HIV infections among young people in Ghana was 1.5% for young women and 0.2% for young men [9]. The prevalence of HIV infections among young people in Ghana is lower than the prevalence in other SSA countries, which may be due to the low uptake of HIV testing services [10]. HIV testing is crucial for the control and management of the disease [11]. It is an entry point for many prevention interventions and an essential strategy for accessing health care and treatment among people living with HIV/AIDS. It also helps in reducing HIV transmission and improving health outcomes [12]. Young people remain at the midpoint of HIV infection, in terms of transmission, vulnerability and impact. Young people will determine the course of the HIV epidemic, making them a priority for HIV/AIDS programmes [13]. Studies have shown that the uptake of HIV testing services among young women was associated with educational status, marital status, multiple sexual partners, sexually transmitted infections, visits to health facilities, alcohol use, living in urban areas and antenatal visits [11, 14]. Also, gender, age at first sex, knowledge of HIV, exposure to mass media, awareness of HIV testing and age were associated with the uptake of HIV testing services among young people [15, 16].

In Ghana, the prevalence of HIV testing among young people is low (less than 50%) [17, 18], which suggest that a majority of young people do not know their HIV status. This poses a challenge to achieving global HIV/AIDS-related targets, including Target 3.3 of the United Nations Sustainable Development Goals (ending HIV as a public health threat by 2030) and the UNAIDS 95-95-95 target, where 95% of persons living with HIV know their status, 95% of persons who know their status are put on sustained antiretroviral therapy and 95% of persons on treatment have their viral load suppressed by 2025 [19]. Currently, in Ghana, 58% of persons infected with HIV know their status, which is far below the 95% target [20]. In the quest to achieve the global targets, the Ghana AIDS Commission and other stakeholders have rolled out some programmes (i.e. Know Your Status, Free-to-Shine and Heart-to-Heart campaigns), which among other things, aim at increasing the uptake of HIV testing services [20]. Therefore, there is a need to assess the effectiveness of these programmes using recent and nationally representative data. Prior studies among young people in Ghana are limited and outdated [18] or focused on specific geographical regions [17]. Therefore, little is known about risky sexual behaviours and HIV testing among young people in Ghana. Findings from this study will help inform sexual health and HIV policies and programmes. The objective of this study was to assess the relationship between engaging in risky sexual behaviours and the uptake of HIV testing services among young people, using data from the 2017/2018 Ghana Multiple Indicator Cluster Survey (MICS), a nationally representative study.

## Methods

### Data source and study design

We obtained the 2017/2018 Ghana MICS data from UNICEF through a formal request. Permission to use the anonymized data was granted by UNICEF. The 2017/2018 MICS collected data on population and health indicators using Computer-Assisted Personal Interviewing (CAPI) across all the previous ten administrative regions of Ghana. The protocols for the 2017/2018 MICS were approved by the Ghana Health Service Ethics Review Committee. Verbal consent was obtained from all adult participants before questionnaires were administered. For participants between the ages of 15–17 years, consent was obtained from their caretakers or parents. All participants were assured of voluntary participation, confidentiality and anonymity of information, as well as the free-will to withdraw from the interview at any point.

### Population and sampling

The target population for the 2017/2018 MICS were Ghanaians between the ages of 15–49 years. A two-stage sampling technique was employed to select the participants. First, 660 enumeration areas/clusters were selected from the 2010 Population and Houses Census list proportional to size. The next stage of the recruitment process involved the selection of 13,202 households using systematic random sampling of which 12,886 were interviewed. Women between the ages of 15–49 years in the selected households were eligible for the women survey. Exactly, 14,609 women were identified in the selected households, of which 14,374 were interviewed, equivalent to a 98.4% response rate. A total number of 5476 men (15–49 years) were subsampled, of which 5323 were interviewed, equivalent to a 97.2% response rate. Data collection was done by trained fieldworkers. Data collection began on 15th October 2017 and ended on 15th January 2018.

### Definition of variables

The main outcome variable of interest in this study was the uptake of HIV testing services. This was a single item (*I don’t want to know the results, but have you ever been tested for HIV?),* originally coded as ‘Yes’ = 1 and ‘No’ = 2 and ‘No response’ = 9. In this study, the outcome variable was recoded as ‘Yes’ = 1 and ‘No’ = 0, and ‘No response’ dropped from the dataset. The covariates included risky sexual behaviours which were measured using three items: age of first sexual intercourse (discrete variable), condom use at last sexual intercourse (‘Yes’ = 1 and ‘No’ = 2, ‘No response’ = 9) and sex with any other man/woman in the last twelve months (‘Yes’ = 1 and ‘No’ = 2, ‘No response’ = 9). Age of first sexual intercourse was recoded as (8–14 years = 1 ‘early sexual debut’ and 15–24 years = 2 ‘late sexual debut’). Socio-demographic factors included: age of respondent, educational status, marital status, household wealth index, region and type of place of residence. Exposure to mass media was assessed using three items: frequency of reading newspapers, listening to a radio and watching television. Details about the 2017/2018 MICS are provided elsewhere [21].

### Statistical analysis

This study focused on persons between the ages of 15–24 years, therefore observations for persons between the ages of 25–49 years were dropped from the dataset prior to the analysis. The target group were young people who had sex in the last 12 months of the survey. In this regard, observations for young people who never had sex and those who did not have sex in the last 12 months and ‘no response’ were dropped from the data files. In all, 12,348 observations were dropped from the women data file and 4621 from the men data file. A weighted sample size of 1702 for young women and 734 for young men were included in the analysis. Data were analysed at three levels, including univariate, bivariate and multivariable. Descriptive statistics such as frequency, percentage, mean and standard deviation were computed at the univariate level, while the Chi-square test was computed at the bivariate level to check for associations between variables. Finally, Binomial Logistic Regression was computed at the multivariable level to identify significant predictors of HIV testing. We adjusted for clustering, stratification and sample weight using the ‘svy’ STATA command. Adjusted odds ratios were reported at the 95% confidence interval and the 0.05 significance level.

## Results

### Descriptive statistics

It was found that the majority of the young women were aged 20–24 years (62%) and 44% had obtained junior high school education. Also, more than half of the young women were unmarried (66%) and resided in rural areas (52%), while 17% were in the poorest wealth index. Regarding exposure to mass media, eight in ten young women did not read newspapers, 33% did not listen to radio, while 20% did not watch television. On the other hand, more than half of the young men were aged 20–24 years and 41% had junior high school education. Eight in ten young men were unmarried, 16% were in the poorest wealth index and 52% lived in rural areas. About eight in ten (78%) young men did not read newspapers, 14% did not listen to radio and 13% did not watch television. Regarding risky sexual behaviours, 7% of the young women had multiple sexual partners, while 79% did use a condom during last sexual intercourse. The prevalence of early sexual debut was 17% and 13% among young women and young men respectively. Exactly 68% of the young men did not use a condom during last sexual intercourse, while 20% had multiple sexual partners. We also found that the majority of the young women (68%) and the young men (87%) had not tested for HIV (Table 1).

**Table 1:** Participants’ characteristics

Characteristic	Young women n (%)	Young men n (%)
*Age (years)*		
15–19	653 (38)	209 (28)
20–24	1049 (62)	525 (72)
*Educational status*		
Pre-primary/no education	95 (6)	26 (4)
Primary	244 (14)	97 (13)
Junior high	752 (44)	301 (41)
Senior high	542 (32)	282 (38)
Higher	69 (4)	28 (4)
*Marital status*		
Currently married/in union	523 (31)	127 (17)
Formerly married/in union	60 (3)	19 (3)
Never married/ in union	1119 (66)	588 (80)
*Wealth index*		
Poorest	288 (17)	115 (16)
Second	354 (21)	136 (19)
Middle	401 (23)	191 (26)
Fourth	378 (22)	174 (24)
Richest	282 (17)	118 (16)
*Area of residence*		
Urban	809 (48)	349 (48)
Rural	892 (52)	384 (52)
*Region*		
Western	195 (11)	72 (10)
Central	170 (10)	50 (7)
Greater Accra	216 (13)	52 (7)
Volta	157 (9)	66 (9)
Eastern	203 (12)	101 (14)
Ashanti	338 (20)	241 (33)
Brong-Ahafo	180 (11)	64 (9)
Northern	171 (10)	50 (7)
Upper East	40 (2)	16 (2)
Upper West	32 (2)	22 (3)
*Frequency of reading newspaper*		
Not at all	1468 (86)	577 (78)
Less than once a week	117 (7)	93 (13)
At least once a week	91 (5)	35 (5)
Almost everyday	26 (2)	29 (4)
*Frequency of listening to the radio*		
Not at all	561 (33)	99 (14)
Less than once a week	318 (19)	118 (16)
At least once a week	407 (24)	170 (23)
Almost everyday	417 (24)	347 (47)
*Frequency of watching television*		
Not at all	345 (20)	95 (13)
Less than once a week	197 (12)	173 (24)
At least once a week	345 (20)	151 (20)
Almost everyday	816 (48)	314 (43)
*Age at first sexual intercourse*		
8–14 years (Early sexual debut)	228 (17)	94 (13)
15–24 years (Late sexual debut)	1413 (83)	639 (87)
*Condom used at last sexual intercourse*		
Yes	363 (21)	233 (32)
No	1339 (79)	501 (68)
*Multiple sexual partners*		
Yes	116 (7)	144 (20)
No	1586 (93)	590 (80)
*Tested for HIV*		
Yes	549 (32)	92 (13)
No	1152 (68)	623 (87)

### Association between risky sexual behaviours and HIV testing among young people

It was found that 30% of young women who had multiple sexual partners had tested for HIV. Exactly 34% of young women who did not use a condom at last sexual intercourse had tested for HIV, while 34% of young women who had early sexual debut had tested for HIV. On the other hand, 12% of young men who had multiple sexual partners had tested for HIV. Similarly, 12% of young men who did not use a condom at last sexual intercourse had tested for HIV, while 7% of young men who had early sexual debut had tested for HIV. There was no statistically significant association between risky sexual behaviours and HIV testing (p > 0.05) (Table 2).

**Table 2:** Chi-square analysis of risky sexual behaviours and HIV testing among young people in Ghana

Risky sexual behaviours	Young women		Young men n (%)	
	Not tested n (%)	Tested n (%)	Not tested n (%)	Tested n (%)
*Age at first sexual intercourse*				
8–14 years (Early sexual debut)	190 (66)	99 (34)	81 (93)	6 (7)
15–24 years (Late sexual debut)	963 (68)	451 (32)	541 (86)	86 (14)
*Condom used at last sexual intercourse*				
Yes	263 (72)	100 (28)	199 (86)	32 (14)
No	890 (66)	449 (34)	423 (88)	60 (12)
*Multiple sexual partners*				
Yes	82 (70)	34 (30)	126 (88)	17 (12)
No	1071 (68)	515 (32)	496 (87)	76 (13)

### Predictors of HIV testing among young people in Ghana

The result showed that both young women (AOR = 2.19; 95% CI 1.56–3.07) and young men (AOR = 3.38; 95% CI 1.18–9.64) aged 20–24 years had a higher likelihood of being tested for HIV compared to those aged 15–19 years. In addition, young women with at least junior high school education (AOR = 2.03; 95% CI 1.08–3.81) were more likely to test for HIV compared with those who had pre-primary or no formal education. Young women who were never married or in a union had 61% of reduced odds of being tested for HIV compared with those who were currently married or in a union. Further, young women in the Northern Region had 48% reduced odds of being tested compared with those from the Western Region. On the other hand, young men with higher education were 6.6 times more likely to be tested compared with those with pre-primary or no formal education (AOR = 6.65; 95% CI 0.42–7.75). Young men in the Central Region (AOR = 6.55; 95% CI 1.72–24.99) and Volta Region (AOR = 4.48; 95% CI 1.22–16.41) had higher likelihoods of being tested compared with those in the Western Region. Also, young men who read a newspaper at least once a week were 4 times more likely to test for HIV compared with those who did not read a newspaper (AOR = 4.01; 95% CI 1.18–13.60). The effect of risky sexual behaviours on HIV testing was not statistically significant (p > 0.05) (Table 3).

**Table 3:** Binomial Logistic Regression analysis of risky sexual behaviour and HIV testing among young people in Ghana

Characteristic	Young women AOR (95% CI)	Young men AOR (95% CI)
*Age (years)*		
15–19	1 (ref)	1 (ref)
20–24	2.19 (1.56–3.07)*	3.38 (1.18–9.64)*
*Educational status*		
Pre-primary or no education	1 (ref)	1 (ref)
Primary	1.44 (0.71–2.93)	0.27 (0.03–1.91)
Junior high	2.03 (1.08–3.81)*	0.50 (0.10–2.35)
Senior high	2.04 (1.06–3.91)*	1.82 (0.42–7.75)
Higher	3.89 (1.65–9.13)*	6.65 (0.42–7.75)*
*Marital status*		
Currently married/in union	1 (ref)	1 (ref)
Formerly married/in union	1.49 (0.69–3.20)	2.03 (0.24–16.89)
Never married/in union	0.39 (0.27–0.56)*	0.67 (0.27–1.64)
*Wealth index*		
Poorest	1 (ref)	1 (ref)
Second	1.05 (0.65–1.70)	0.50 (0.15–1.67)
Middle	1.13 (0.65–1.97)	1.32 (0.44–3.92)
Fourth	1.73 (0.96–3.13)	0.76 (0.17–3.42)
Richest	1.10 (0.59–2.07)	0.55 (0.11–2.81)
*Area of residence*		
Urban	1 (ref)	1 (ref)
Rural	1.08 (0.78–1.50)	1.39 (0.64–3.03)
*Region*		
Western	1 (ref)	1 (ref)
Central	0.72 (0.43–1.21)	6.55 (1.72–24.99)*
Greater Accra	0.80 (0.46–1.40)	3.29 (0.77–13.89)
Volta	0.70 (0.39–1.26)	4.48 (1.22–16.41)*
Eastern	0.76 (0.42–1.36)	2.42 (0.62–9.40)
Ashanti	0.65 (0.39–1.09)	0.39 (0.10–1.53)
Brong-Ahafo	1.13 (0.64–1.97)	1.18 (0.27–5.13)
Northern	0.52 (0.29–0.92)*	0.72 (0.18–2.79)
Upper East	0.66 (0.35–1.27)	1.29 (0.20–8.19)
Upper West	0.71 (0.39–1.30)	0.51 (0.09–2.72)
*Frequency of reading newspaper*		
Not at all	1 (ref)	1 (ref)
Less than once a week	1.20 (0.74–1.97)	1.92 (0.66–5.59)
At least once a week	1.21 (0.59–2.46)	4.01 (1.18–13.60)*
Almost everyday	1.74 (0.48–6.26)	2.57 (0.67–9.88)
*Frequency of listening to the radio*		
Not at all	1 (ref)	1 (ref)
Less than once a week	0.63 (0.42–0.97)*	3.44 (0.92–12.85)
At least once a week	0.82 (0.56–1.21)	2.21 (0.59–8.23)
Almost everyday	1.17 (0.81–1.67)	2.22 (0.68–7.24)
*Frequency of watching television*		
Not at all	1 (ref)	1 (ref)
Less than once a week	0.75 (0.44–1.28)	0.72 (0.23–2.30)
At least once a week	0.90 (0.57–1.42)	0.44 (0.15–1.28)
Almost everyday	1.17 (0.77–1.78)	0.48 (0.16–1.38)
*Age at first sexual intercourse*		
8–14 years (Early sexual debut)	1 (ref)	1 (ref)
15–24 years (Late sexual debut)	0.80 (0.54–1.20)	1.73 (0.56–5.36)
*Condom used at last sexual intercourse*		
Yes	1 (ref)	1 (ref)
No	1.17 (0.80–1.69)	0.69 (0.33–1.43)
*Multiple sexual partners*		
Yes	1 (ref)	1 (ref)
No	0.99 (0.54–1.81)	1.29 (0.57–2.94)

^*^p-value < 0.05

## Discussion

The prevalence of HIV testing was 32% among young women and 13% among young men. This implies that there was low uptake of HIV testing services among young people. Meaning that many young people do not know their HIV status, posing a threat to achieving Target 3.3 of the SDGs and the UNAIDS 95-95-95 target. This finding is supported by previous studies [11, 14]. For instance, a similar study among young people in South Africa revealed that 32.7% and 17.7% of young women and young men respectively had tested for HIV [22]. The prevalence in this study is higher than findings from a similar survey that was conducted a decade ago in the country (20% for young women and 9% for young men) [18]. This finding suggests that the proportion of young people who utilize HIV testing services has increased over time. The differences in findings may be explained by the implementation of HIV/AIDS and youth-friendly programmes over the last decade. For example, the Ghana Adolescent Health and Development programme received huge financial support from the UKAID through the Palladium Group in 2015. Through that support, more youth-friendly clinics were established across the country coupled with the refurbishing of old youth-friendly clinics and the training of youth-friendly health providers to provide age-appropriate health services, including voluntary HIV counselling and testing. Also, the Ghana AIDS Commission rolled out a number of campaigns, including the Heart-to-Heart and Free-to-Shine campaigns which among other things seek to increase the utilization of HIV testing services among persons living in Ghana [20].

We also found that young women were more likely to test for HIV compared with young men. A similar study among youth in four sub-Saharan Africa countries (Congo, Uganda, Nigeria and Mozambique) revealed that young men had lesser odds of being tested for HIV compared with young women [15]. Further, evidence shows that women are more likely to get tested for HIV than men [23, 24]. In Ghana, voluntary HIV counselling and testing services have been integrated into antenatal care as part of measures to reduce perinatal HIV transmission [25]. Perhaps some of the young women in this study accessed antenatal care in the past, hence may explain the differences in the uptake of HIV testing services across gender. The uptake of HIV testing services was influenced by educational status. Young people with higher education were more likely to test for HIV compared with those who had no formal education. This finding has been supported by prior studies [11, 14]. For example, a study in Nigeria revealed that young people with senior high school education were two times more likely to have tested for HIV compared with those with no education [26]. In addition, a survey in Tanzania revealed that young women with higher education had higher odds of being tested for HIV [11]. These findings are understandable because women with higher education can access and comprehend information about HIV infection, transmission and prevention. Also, highly educated women are more likely to be employed, hence they can afford HIV testing services. More importantly, education empowers women to make informed health-seeking decisions [14].

The findings of this study also revealed that married or in union women had higher chances of being tested for HIV compared with unmarried women. This finding is expected, because some faith-based organizations in Ghana recommend that potential couples get tested for HIV before marriage. This practices by faith-based organizations may explain why married young women were more likely to have tested for HIV. In addition, married women are more likely to access antenatal care. Further, young people between the ages of 20–24 years had higher likelihoods of being tested compared with those aged 15–19 years. This finding has been confirmed by a previous study in Techiman, suburb of Ghana, where a higher proportion of young people aged 20–24 years had tested for HIV compared with young people aged 15–19 years [17]. A possible reason is that young people aged 15–19 years (adolescents) face many barriers when accessing healthcare services. Some of these barriers include judgemental attitude of health providers, lack of privacy and confidentiality, financial constraints and inadequate information about testing centres [27]. In addition, the uptake of HIV testing services among young people was determined by geographical region. Young women in the Northern region were less likely to test for HIV, while young men in the Volta and Central regions were more likely to test for HIV. A similar study conducted in South African revealed geographical inequities in the uptake of HIV testing services among young people [22]. In Ghana, there is an inequitable distribution of health care resources across regions. A considerable proportion of the health facilities and health workers are in the southern part of Ghana [28]. Further, young people exposed to mass media were more likely to be tested for HIV. These findings are expected, because young people exposed to mass media may have access to information on HIV transmission and prevention. Evidence shows that young people who are knowledgeable about HIV are more likely to utilize HIV testing services [29].

The result also showed that young people engaged in risky sexual behaviours, with the majority engaging in unprotected sex. Young people who engage in risky sexual behaviours are predisposed to HIV infections. A cross-sectional study in the Greater Accra region revealed that condom use among young people was low [17]. This finding was confirmed by another study in Ethiopia, where more than half of adolescents did not use a condom during sexual intercourse [7]. We found no significant association between engaging in risky sexual behaviours and the uptake of HIV testing services. Similar studies also found no association between risky sexual behaviours and HIV testing [12, 30]. On the contrary, a study in Ethiopia revealed that young women who had multiple sexual partners were more likely to have tested for HIV [14]. The differences in findings may be attributed to disparities in contextual factors.

### Implications and recommendations

The findings from this study have implications for HIV policy and programming. The low use of condoms and HIV testing services among young people are matters of public health concern and require the attention of stakeholders. The findings imply that young people in Ghana are at a higher risk of HIV infection, yet they are unaware of their HIV status. These challenges pose threats to HIV prevention and treatment and may derail Ghana’s efforts towards ending HIV as a public health threat by 2030. Moreover, young people who are unaware of their HIV status may be living with the disease unknowingly, hence they may transmit the virus to uninfected persons. The adverse effects of late detection of HIV cannot be overemphasized. It can lead to complications, poor health outcomes, disability and mortality. Since young people will determine the course of HIV in the future [13], stakeholders, such as the Ghana AIDS Commission, Ministry of Health and Non-Governmental Organizations, must prioritize vulnerable young people in HIV intervention. Promoting formal education among young people will help increase the uptake of HIV testing services. In addition, stakeholders can leverage the mass media to disseminate information about HIV testing services to young people. There is also a need to educate young people about the consequences of risky sexual behaviours as well as promote condom use. This study provides relevant information for HIV prevention and control, yet it is not devoid of limitations. Surveys cannot explain the many intricate views of participants regarding a subject matter; therefore, the findings should be interpreted with caution. Notwithstanding, the findings from this study can be generalized to the population because the data was nationally represented.

## Conclusion

This study demonstrated that the majority of the young people engaged in unprotected sexual intercourse and more than half had not tested for HIV. There was no significant association between engaging in risky sexual behaviours and the uptake of HIV testing services. The uptake of HIV testing was influenced by older age, higher education and being married. There were regional disparities in the uptake of HIV testing services. Exposure to mass media was an enabling factor for utilization of HIV testing services. Going forward, HIV prevention interventions should prioritize young people who are unmarried, less educated and younger. Stakeholders can leverage the mass media to promote sex education and utilization of HIV testing services among young people.

## Data Availability

The data used in this study is owned by UNICEF, therefore, the authors cannot share the data. Interested persons can contact UNICEF for the data (https://mics.unicef.org/surveys). The authors confirm they did not have any special access or privileges to the data that other researchers would not have.

## Abbreviations

MICS: Multiple Indicator Cluster Survey
HIV: Human Immunodeficiency Virus
AIDS: Acquired Immuno-Deficiency Syndrome
UNICEF: United Nations International Children’s Emergency Fund
CAPI: Computer Assisted Personal Interviewing
SDG: Sustainable Development Goal
SSA: Sub-Sahara Africa
